# The use of coaching to enhance students' learning, self‐efficacy and performance in endodontics

**DOI:** 10.1111/iej.14241

**Published:** 2025-04-19

**Authors:** Kathryn Fox, Fadi Jarad, Annemarie Baaij

**Affiliations:** ^1^ School of Dentistry, Institute of Life Course and Medical Sciences, Faculty of Health and Life Sciences University of Liverpool Liverpool UK; ^2^ Academic Centre for Dentistry Amsterdam (ACTA) University of Amsterdam and Vrije Universiteit Amsterdam Amsterdam The Netherlands

**Keywords:** coaching, education, endodontology, postgraduate, undergraduate

## Abstract

The positive effects of coaching on enhancing performance and development within sport have been well recognized over the past 40 years. More recently, the beneficial effects of utilizing a coaching approach upon learning have been recognized in the commercial world and, increasingly, in education. The potential of using a coaching approach within medical education has been investigated, with observed improvements in performance, academic success, resilience, professional identity and self‐efficacy – all of which are important in the development of clinicians. Endodontic education and training, whether undergraduate or postgraduate, aim to prepare clinicians to undertake high‐quality endodontic care for patients. To achieve this, the student needs to have the capability to perform the appropriate level of endodontic treatments, but also the confidence and self‐efficacy to execute those treatments, where self‐efficacy contributes to how successful their performances will be. This paper outlines the sources and development of self‐efficacy and describes how a coaching approach can be used to enhance students' skills acquisition and performance, both during clinical teaching sessions and in their periodic development reviews.

## INTRODUCTION

The aim of education in endodontics – both undergraduate and postgraduate – is for students to gain the necessary knowledge, practical skills and deep understanding to provide good quality endodontic care at graduation. To achieve this, students also need to have self‐efficacy; the individual belief in their capacity to perform specific tasks in a given setting successfully in order to reach their goals (American Psychological Association, 2024; Bandura, 1977). Therefore, students must both believe in their endodontic capabilities and have an accurate awareness of their own competency level. Although new dental graduates may not be expected to be able to provide the full range of endodontic treatments, they should ensure that their patients receive appropriate endodontic care (Baaij et al., 2024) by recognizing when to appropriately refer patients who require care outside their existing skillset.

The positive effects of coaching in enhancing performance and development in a variety of fields have been recognized (Bozer & Jones, 2018; Theeboom et al., 2014), including specific workplace skills and more generic capabilities such as communication, time management and teamworking. Coaching has been shown to increase self‐awareness, self‐efficacy, self‐regulation, goal setting, attainment and resilience (Bozer & Jones, 2018; Grant, 2003; Moen & Allgood, 2009; Richardson et al., 2023; Wang et al., 2021). Within higher education, coaching interventions have also been shown to benefit students through academic, emotional and psychological support and in professional development (Shorey et al., 2022), as well as improving student engagement and retention (Baker & Bettinger, 2011).

Alongside the general benefits to professional development of a student developing self‐efficacy, the gaining and refinement of skills in endodontics by undergraduate and postgraduate students can also be enhanced by faculty employing coaching approaches in their teaching and assessment.

## WHAT IS COACHING?

Coaching has been described as ‘a tool to help learners achieve their fullest potential by highlighting insights into their own assumptions, perceptions and behaviours’ (Hammoud et al., 2024). van Nieuwerburgh (2012) defined coaching in education as a one to one conversation ‘where the coach facilitates the self‐directed learning’ of the student ‘through questioning, active listening, and appropriate challenge in a supportive and encouraging climate’ and also highlighted how coaching enhances the development of self‐awareness and personal responsibility of the student. Coaching is often confused with mentoring and, although there is some overlap between the two, mentoring focuses on a more‐experienced individual providing specific advice and guidance to a student, whereas coaching facilitates the student's self‐awareness and self‐directed learning (Starr, 2021).

## DEVELOPMENT OF SELF‐EFFICACY

**FIGURE 1:**
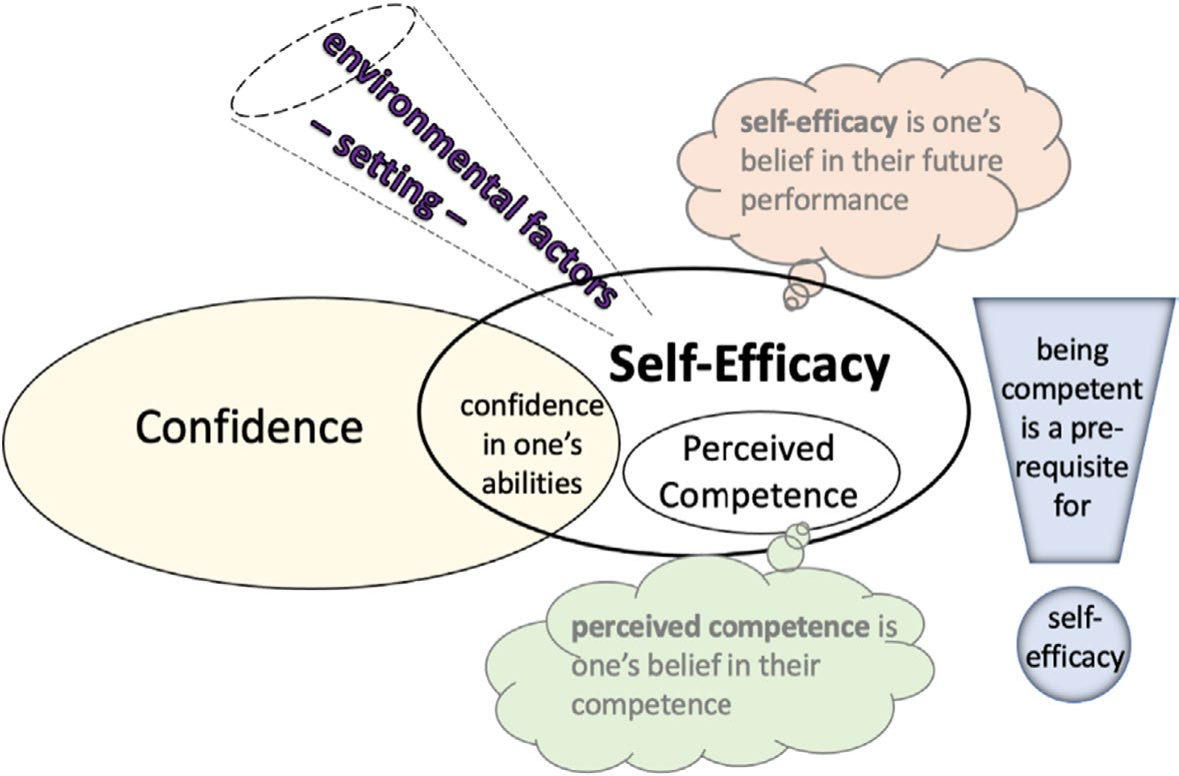
Graphical presentation of how the concepts of confidence, self‐efficacy and perceived competence relate to each other (ellipses are not necessarily to scale). Adapted from Baaij (2023).

By the end of their training, undergraduate or postgraduate students should be both skilled and empowered to perform endodontic treatments to the appropriate level. The achievement of competence has been defined as when ‘individuals are capable of practicing independently and of assuming responsibility for their continued professional growth’ (Chambers, 1998). However, whether a student will actually use their developed clinical competencies, and the level at which they perform, can be predicted by their self‐efficacy (Gist & Mitchell, 1992; Pajares & Miller, 1994; Zimmerman, 2000). Self‐efficacy combines confidence in ability with perceived competence (Figure 1) (Baaij, 2023), and it can also be influenced by external factors such as peer‐learning and supervisor feedback.

Bandura (1977) has stated that the development of self‐efficacy is influenced by four primary sources:

1. Performance accomplishments (PA) – gained from the personal experiences of undertaking a task successfully. Within endodontics this could be the progression from simulation to increasingly complex patient care (Baaij, 2023) as positive experiences are gained (Baaij et al., 2020, 2021; Bandura, 1977; Bandura & Schunk, 1981).
2. Vicarious experience (VE) – that is, learning by observing others performing similar tasks (Stroben et al., 2016). This can also occur when students present and discuss their clinical work with their peers (Baaij, 2023).
3. Verbal persuasion (VP). In this case, a student receives positive verbal feedback which persuades them that they possess the skills and capabilities to be successful (Bandura, 1977). This is most effective when it comes from a trusted, credible source such as a respected teacher of endodontics (Won et al., 2017). Therefore, the encouragement or discouragement given during or after a clinical session can affect the development of the learner's self‐efficacy (Wulf et al., 2010).
4. Physiological and emotional states (PS) – Haidt (2024) noted that stress and anxiety, which have significantly increased in young people, can undermine self‐efficacy, and it has been proposed that the identification and management of psychosocial stress should therefore be incorporated into dental curricula (Dawson et al., 2024; Haug et al., 2021).

Individuals with low self‐efficacy tend to attribute failure to their lack of capabilities, even when this is incorrect (Bandura, 1977; Gist & Mitchell, 1992; Leganger et al., 2000), whereas those with high self‐efficacy will more readily undertake challenging tasks, demonstrate perseverance and perform better in stressful situations (Bandura, 1977, 2006; Zimmerman, 2000).

## DEVELOPMENT OF PROFESSIONAL COACHING

To understand how adopting a coaching approach can benefit students of endodontics, it is worth looking at how this has been applied elsewhere. Coaching has been commonplace in professional sports for many years. In the 1970's Tim Gallwey, a top American tennis player, realized that, during his coaching sessions, he was focusing more on the process of teaching, rather than whether his students were learning. He noted that his teaching approach was often ineffective because a player's (in our case student's) own ‘internal critic’ got in the way (Gallwey, 1974). Gallwey went on to describe this as the ‘Outer and Inner Games’; competing in the ‘outer game’ with the physical opponent, but also with themselves (the ‘inner game’), where nervousness, self‐deprecation and fear of failure was an obstacle to peak performance. Consequently, he proposed that a person's performance in any activity is equal to their innate potential minus their internal interference:

Performance=Potential−Interference

As a result, Gallwey developed a whole new style of coaching underpinned by clarifying goals, establishing motivation, raising self‐awareness and increasing self‐efficacy. He also focused the learners' full attention on external aspects (e.g., the position of the ball, the position of their feet or the sound of the racquet), enabling the athletes to become fully absorbed in the moment and remove the learner's internal interferences regarding achieving success or failure. This coaching style increased learning, enjoyment and improved overall performance (Gallwey, 1974) and has been subsequently applied successfully to other sports and music performance (Mouton, 2016).

The benefits of such coaching techniques were noticed subsequently by the corporate world and this model of coaching is now recognized worldwide (Whitmore, 2017). Over the past 20 years, there has also been a substantial increase in the study of coaching activity and its proven efficacy within the educational sector (van Nieuwerburgh, 2012) and therefore it would seem appropriate to investigate the value of adopting a coaching approach within endodontic education.

## DEVELOPMENT OF COACHING IN MEDICAL EDUCATION

Unlike athletes, traditional concepts in clinical training have assumed that, after achieving competency, surgeons no longer need any assistance (Gawande, 2011). Within the medical field, the benefits of coaching were first highlighted by Gawande, a surgeon, who, in trying to improve his tennis serve, was not given instructions as to what to do or change, but was instead encouraged by the coach to become aware of his body positioning and the effect that this had on the resulting outcome. In doing so, he realized that his serve was consistently improving and subsequently recognized the potential parallels between the physical performance of professional athletes and surgeons (Gawande, 2011). This led to a significant interest in coaching within the medical field to improve surgical performance (Wolff et al., 2020) and provide individualized learner‐centred support to students (Richardson et al., 2024). It was also noted that this increased the student clinician's self‐determination and self‐efficacy, resulting in decreased burnout (Deiorio et al., 2016; Gazelle et al., 2015). Research has demonstrated the positive effects of solution‐focussed coaching in medical education, not only on academic success and professional identity but also in building resilience (Lovell, 2018), flexibility (Maini et al., 2020) and overall wellbeing (Dyrbye et al., 2019) – areas of particular importance to health professionals in the current uncertain climate. These beneficial outcomes would also be expected to apply equally to endodontic education. However, to date, no published investigations specifically address the use of coaching in endodontics, and there are no recommended models. This paper aims to propose a model demonstrating how an innovative coaching approach could enhance the endodontic education of both undergraduates and postgraduates by increasing their self‐efficacy and empowering students to take personal responsibility for their learning.

## AREAS OF COACHING

Coaching can be grouped into 2 main areas (Cox et al., 2018);

- Skills and performance coaching
- Development coaching

Although these are often described as separate entities, it has been suggested that there is an evolution in coaching, going from specific skills coaching through more generalized performance coaching to, finally, personal or professional development coaching (Jackson & Cox, 2018). This mirrors the development of self‐efficacy in endodontics, where students often learn individual technical skills in a pre‐clinical setting, perform them subsequently on a patient in a clinical setting, and finally integrate this into their own developing professional identity as a clinician. The two areas of coaching (above) that benefit the student have also been described as:

- Coaching in the Moment – daily interactions focusing on improving the student's performance and skills during pre‐clinical/clinical practice, and
- Coaching over Time – longitudinal relationships outside the clinical setting that promote the student's reflection on their overall progress and set developmental goals (Landreville et al., 2019)

As medical and dental (including endodontic) education have moved towards competency‐based, longitudinal assessment, the data collected regarding the student's performance has increased. These large data sets can be difficult for the student to assimilate and use when agreeing learning goals. Therefore, the Royal College of Physicians Surgeons of Canada suggested the use of a ‘coach’ to create an educational alliance with the student – where the coach and student are working towards a common goal (Richardson et al., 2024). This has been endorsed by the American Medical Association (AMA) and the Accreditation Council for Graduate Medical Education (ACGME), who have both proposed that coaching should be integrated into all medical education programmes (Stanford Medicine, 2024).

## COACHING RELATIONSHIP – TRUST AND PSYCHOLOGICAL SAFETY

For coaching to be successful, the relationship between the coach and student must be one with absolute trust. If this is not achieved, often due to the inherent hierarchical relationships within education, the student will be unable to express any vulnerability in terms of perceived weaknesses, and their internal defence mechanisms will be apparent (Parsons et al., 2021). Trust is enhanced when the role of the coach and the expectations and goals of the coaching process are clarified (Jones & Andrews, 2019) and an environment of psychological safety is created (Edmondson, 1999).

**FIGURE 2:**
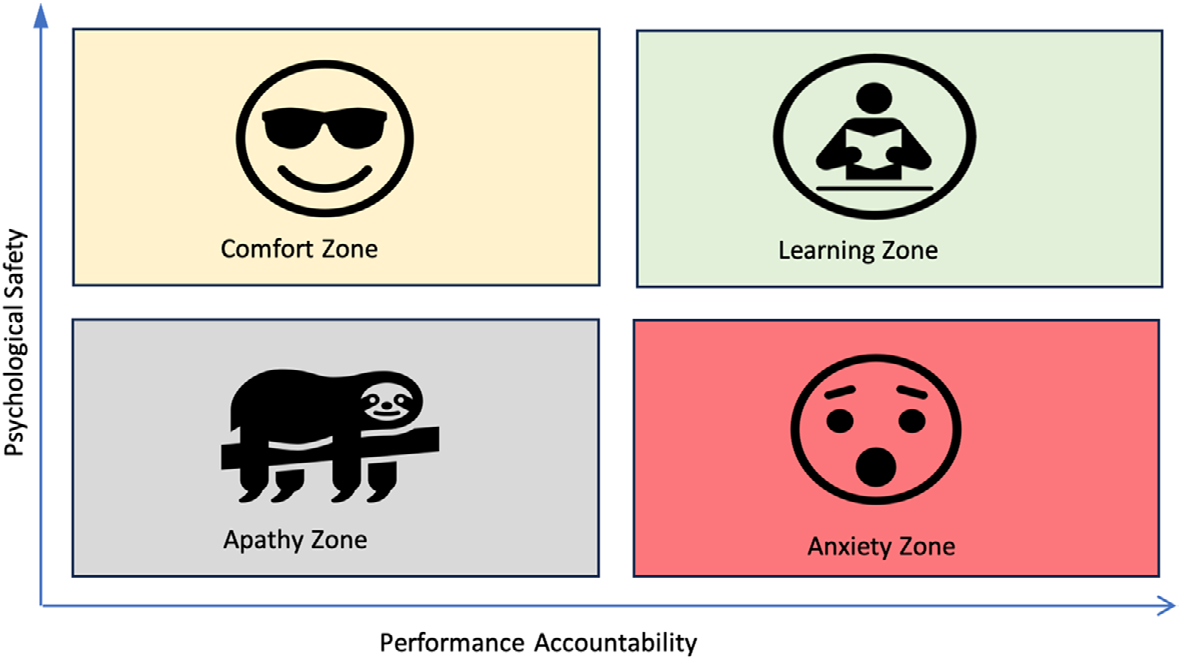
The effects of psychological safety vs. performance accountability on learning. Adapted from Edmondson (2012).

It should be noted that psychological safety is not about being ‘nice’ or lowering standards; it is about producing the right conditions to allow learning and progress to take place. If psychological safety is present but performance accountability is low, the student will remain in their comfort zone and not make the desired progress. Alternatively, if the accountability is high but the psychological safety is low, the environment generates anxiety and fear of making mistakes. Therefore, to achieve maximum learning and peak performance in endodontics, both psychological safety and performance accountability are required (Figure 2).

In a situation where an individual has the dual role of supervisor and coach, there could be a perceived conflict of interest (and undermining of trust). Therefore, it has been recommended that faculty with coaching roles should be distinctly separate from those in supervisory roles and not make progress decisions (Baenziger et al., 2023). However, this may not be easy to accommodate within an institution where there may be a limited number of supervising staff. In such circumstances, a ‘four‐eyed approach’, that is, a 2nd assessor, could help reduce the perceived conflict.

Within endodontic teaching, the two aspects of coaching described above should be considered; first, using a coaching approach to deliver feedback, by the endodontic supervisor ‘in the moment’ – where the aim is to improve the student's specific skills (such as access cavities) – and this use of a coaching approach within dentistry has recently been described (Roberts et al., 2022). Secondly, using developmental coaching ‘over‐time’ where a purely nonjudgemental approach is delivered by a coach, away from the chairside, who has no direct influence on the student's progress. As yet this type of coaching has only been reported in medicine (Richardson et al., 2024) but it should be highlighted, that subject‐specific knowledge (i.e., the coach having a dental background) would still be advantageous to help the student reflect fully and understand the context behind any clinical issues raised.

Providing ‘in the moment’ and ‘over time’ feedback provides an ideal opportunity for the use of ‘verbal persuasion’ as a source of self‐efficacy. Feedback that is delivered well, on the student's performance in endodontics, can be transformative. However, feedback delivered poorly can feel devastating, and it is the recipient's *reaction* to the feedback, rather than the content of the feedback itself, that determines their subsequent performance (Barends et al., 2022). To avoid this, the recipient of the feedback needs to feel that the supervisor not only has high standards, but that the recipient can meet these, and be supported in doing so – that is, the supervisor has a ‘mentor mindset’ (Yeager, 2024). Coaching in this manner implements the characteristics of a ‘Master Adaptive Learner’ process, where the student identifies the gaps in their own skills, selects suitable learning strategies and subsequently plans, learns, assesses and adjusts their own learning as required (Hammoud et al., 2024).

## PRACTICAL STEPS FOR CLINICAL EDUCATORS IN ENDODONTICS – THE GROW COACHING MODEL

In order to facilitate coaching conversations, the GROW model was developed (Whitmore, 2017). Each phase of the GROW model has a distinct purpose.

*G*oal (What do you want to get out of this? – Establishes the outcome wanted from the coaching).

*R*eality (Where are you now? – clarifies the current situation).

*O*ptions (What could you do? – shifts to a solution‐focussed conversation).

*W*ill (What will you do? – determines commitment to action).

Although other frameworks have been proposed and utilized in coaching they all have a common theme – in that they are utilized to clarify the individual's goals, facilitate the exploration of potential solutions, and empower the student to take personal responsibility for the action taken (Carey et al., 2011). The GROW model is recognized globally and can serve as a useful framework to structure clinical teaching in endodontics. The most straightforward way to introduce coaching into an endodontic program is to use a ‘coaching in the moment’ approach during a clinical teaching session.

## HOW A COACHING APPROACH CAN BE USED TO SUPPORT STUDENT SELF‐EFFICACY IN ENDODONTICS?

### Coaching in the moment

Within an endodontic teaching session, the GROW model can be used to have coaching conversations, both at the start, during and at the end of the session and can be used to facilitate awareness of the 4 sources of self‐efficacy (PA, VE, VP and PS) (Bandura, 1977).

### Start of the session

When the student faces a novel and more challenging clinical situation, a coaching conversation that promotes reflection on previous successes and the understanding gained from these mastery experiences can occur. In addition, discussing vicarious experiences that the student has witnessed (e.g., when assisting their peers) can prove beneficial. Furthermore, acknowledging the stressful nature of undertaking invasive endodontic procedures and discussing where specific support may be required can be helpful for the student (Table 1a).

**TABLE 1:** (a, b). The use of the GROW model at the start and the end of a clinical session (Whitmore, 2017).

Grow	Reality	Options	Will
(a) Start of the clinical session			
What do you want to get out of this session?	Where are you now?	What could you do?	What will you do?
*Clarify goals & criteria for a good performance in endodontics* *Break goals down* *Agree on areas that student would like to receive specific feedback*	*Self‐assessment of previous experiences, similar success in comparable cases (PA)* *Observed experiences of peers (VE)* *Coping in previous stressful situations (PA, PS)*	*Discuss options for treatment. What complications might occur during the endodontic case? How they have been dealt with previously and how to pre‐empt them (PA)* *Discuss options to deal with their own anxiety (PS)*	*Agree plan of action and level of support required (PS)*
(b) End of the clinical session			
What did you want to get out of this session?	Where are you now?	What could you do?	What will you do?
*Review intended goals and criteria for success for the endodontic case*	*Student self‐assessment of own performance during the endodontic case, starting with strengths followed by areas for learning (PA)* *Nonjudgemental dialogue with supervisor focusing on increasing their awareness & their capabilities (VP)*	*Ask what they could do differently next time, what they have learned and what extra resources they might use in future*. *(PA, PS)* *Dialogue to inspire belief in future capabilities in endodontic management (VP)*	*Ask student to commit what will they do, to enhance learning in endodontics (knowledge, skills, professionalism)* *Ask when and how, to encourage responsibility and accountability*

Abbreviations: PA, performance accomplishments; PS, physiological & emotional states (Bandura, 1977); VE, vicarious experience; VP, verbal persuasion.

Throughout the session, the endodontic clinical supervisor should focus on increasing the student awareness of their actions to allow them to self‐correct (for example: ‘Have you noticed the effect of the angle of your bur on the shape of your access cavity?’) rather than offering judgemental advice. This will both facilitate the reduction of their ‘internal interference’ (Gallwey, 1974) and lead to better performance.

### End of the session

At the end of the clinical session, a further coaching conversation starts with reviewing the goals and previously agreed success criteria, followed by asking the students to self‐assess their performance against these goals – both in terms of what went well and what did not reach the intended outcome (Table 1b). Rather than informing the student what they should have done differently, this coaching approach – of raising awareness and personal responsibility – will increase the student's learning (Whitmore, 2017).

### Coaching over time

When endodontic teaching staff are familiar with coaching techniques, ‘coaching over time’ would also be a useful framework to use when reviewing an endodontic portfolio of assessment data from multiple clinical and academic observations with a student, away from the clinical environment. This will facilitate the student's reflection on their present endodontic capabilities, the progress they have made and enable the student to set personal, action‐orientated, goals for future learning; for example, against the list of capabilities outlined in the ESE undergraduate curriculum guidelines (Baaij et al., 2024) and/or their specific national accreditation requirements. By also focusing on the 4 sources that influence self‐efficacy, the student's determination and courage, to take on more complex cases in future, will improve.

**FIGURE 3:**
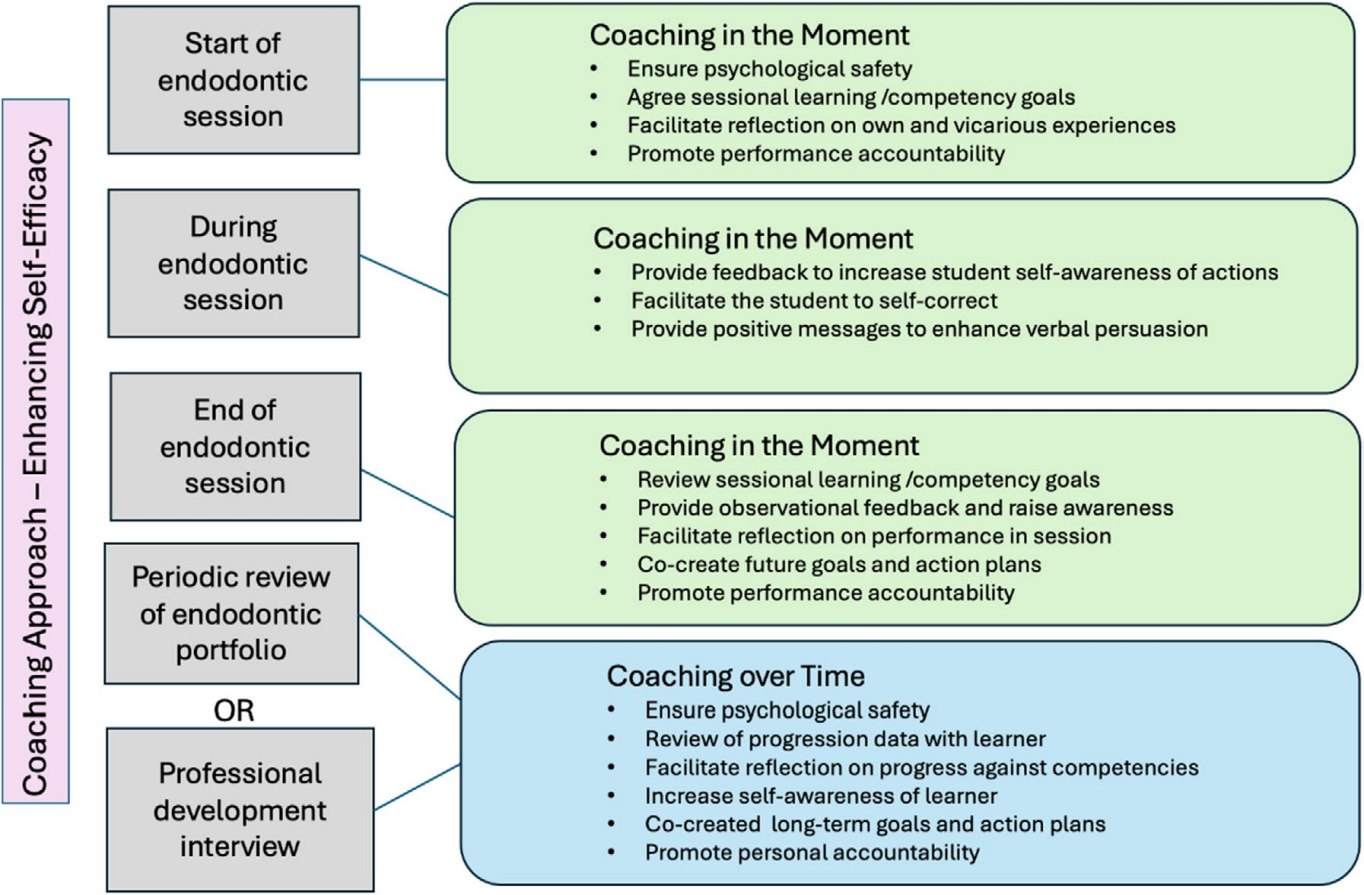
Overview of where a coaching approach could be utilized to enhance self‐efficacy during an endodontic clinical or pre‐clinical session, or as part of an ongoing review of endodontic progress.

Using the GROW framework the coach can provide feedback to increase the student's overall self‐awareness and highlight what may be ‘blind‐spots’ for them, enabling the student to self‐regulate their learning and be accountable for the actions taken. The learning skills and attitude acquired by such an approach can facilitate continuing ongoing professional development, including after graduation (Figure 3).

## CHALLENGES OF INCORPORATING A COACHING CULTURE AND POTENTIAL SOLUTIONS

Introducing a coaching culture into an established dental program can be challenging, and, as yet, this has been implemented fully in only a relatively small number of medical, rather than dental, schools (King et al., 2022). People often resist change, especially if they feel that they do not have the training or skills to implement a new system. Clear communication with the faculty is essential to explain the benefits of a coaching culture – not only for the students, but also in the professional development of the staff, where training to become a coach will benefit the clinician's own personal development (Mukherjee et al., 2024). Coaching to improve the self‐efficacy of the staff, both in their clinical and academic roles, will also, in turn, result in better role‐modelling for the students (Cruess et al., 2008; Passi et al., 2010). Conducting research on the optimal implementation methods within a dental school and their impact on student learning would be valuable, as a comprehensive, whole‐school approach may be ultimately more effective than focusing solely on a single specialty.

For the coaching culture to become embedded and effective, it requires the full support of the senior executive team who must be committed to the processes involved. This includes implementation of training programs, integrated into the existing schedule in a manner that minimizes disruption to reduce curricular congestion, with the longer‐term benefit that empowering students to take more responsibility and accountability for their learning not only improves their development, but also facilitates their independence and reduces reliance on faculty staff.

## CONCLUSIONS

Incorporating coaching into endodontic training offers a potentially transformative approach that could enhance both pregraduate and postgraduate students' self‐efficacy and learning and also foster a supportive learning environment. Furthermore, incorporating a coaching culture across an entire institution would provide benefits to both students and faculty by promoting continuous professional development, collaboration and a proactive and thriving academic community.

## AUTHOR CONTRIBUTIONS

This paper was conceived by Fadi Jarad and Kathryn Fox. Kathryn Fox and Annemarie Baaij prepared the original draft. All three authors edited and revised the manuscript and approved the final version.

## CONFLICT OF INTEREST STATEMENT

There is no conflict of interest to be declared.

## ETHICS STATEMENT

This paper did not include animal or human subjects.

## Data Availability

Data sharing is not applicable to this article as no new data were created or analyzed in this study.
